# A New Nonfullerene Acceptor with Near Infrared Absorption for High Performance Ternary‐Blend Organic Solar Cells with Efficiency over 13%

**DOI:** 10.1002/advs.201800307

**Published:** 2018-03-25

**Authors:** Huan‐Huan Gao, Yanna Sun, Xiangjian Wan, Xin Ke, Huanran Feng, Bin Kan, Yanbo Wang, Yamin Zhang, Chenxi Li, Yongsheng Chen

**Affiliations:** ^1^ State Key Laboratory and Institute of Elemento‐Organic Chemistry Centre of Nanoscale Science and Technology and Key Laboratory of Functional Polymer Materials College of Chemistry Nankai University Tianjin 300071 China

**Keywords:** nonfullerene acceptors, organic solar cells, ternary blends

## Abstract

A new acceptor–donor–acceptor (A–D–A) type nonfullerene acceptor, **3TT‐FIC**, which has three fused thieno[3,2‐b]thiophene as the central core and difluoro substituted indanone as the end groups, is designed and synthesized. **3TT‐FIC** exhibits broad and strong absorption with extended onset absorption to 995 nm and a low optical bandgap of 1.25 eV. The binary device based on **3TT‐FIC** and the polymer **PTB7‐Th** exhibits a power conversion efficiency (PCE) of 12.21% with a high short circuit current density (   *J*_sc_) of 25.89 mA cm^−2^. To fine‐tune the morphology and make full use of the visible region sunlight, phenyl‐C_71_‐butyricacid‐methyl ester (**PC_71_BM**) is used as the third component to fabricate ternary devices. In contrast to the binary devices, the ternary blend organic solar cells show significantly enhanced EQE ranging from 300 to 700 nm and thus an improved * J*_sc_ with a high value of 27.73 mA cm^−2^. A high PCE with a value of 13.54% is achieved for the ternary devices, which is one of the highest efficiencies in single junction organic solar cells reported to date. The results provide valuable insight for the ternary devices in which the external quantum efficiency (EQE) induced by the third component is evidently observed and directly contributed to the enhancement of the device efficiency.

In the past decades, organic solar cells (OSCs) have drawn extensive attentions due to their advantages such as low cost and large area printing production, flexibility, semitransparency, etc.^1^, ^2^, ^3^, ^4^, ^5^, ^6^ Presently, OSCs in labs have made significant progress with power conversion efficiency (PCE) over 13% for single junction devices,^7^ mainly due to new active materials development and device optimizations.^8^, ^9^, ^10^, ^11^ Despite the great success for the fullerene‐based OSCs in earlier years,^12^, ^13^, ^14^ the dominated acceptor materials, fullerene derivatives, have some intrinsic drawbacks such as weak absorption, poor chemical and electronic adjustability, etc., limiting the further improvement of the photovoltaic performance. To address those issues and further improve OSCs efficiencies, great attentions have been paid to develop nonfullerene acceptors (NFAs) with wide and tunnable absorption windows, easily tuned energy levels.^15^, ^16^, ^17^ In just recent years, NFAs‐based OSCs have made significant progress with breakthrough PCEs over 13% for single junction devices.^7^ On the other hand, besides exploring the device optimization methods such as thermal and solvent annealing, additive, etc., the device structures such as tandem^18^, ^19^, ^20^ and ternary devices^21^, ^22^, ^23^, ^24^ have also been explored intensively in order to further improve the performance. In contrast to the complicated fabrication process of tandem structure OSCs, ternary devices with three components (two donors with one acceptor or one donor with two acceptors) have a simpler process to fabricate devices. Furthermore, it can also take the advantage of tandem devices with enhanced photon harvesting by incorporating the third component.^25^, ^26^, ^27^, ^28^, ^29^

In ternary devices, the additional component usually exhibits complementary absorption with the binary materials. Also it needs to have suitable energy levels to offer enough driving forces for exciton dissociation and charge extraction.^21^, ^30^ This is because such a cascade energy structure would bridge the donor and acceptor energy offset and facilitate exciton dissociation and then improve the open‐circuit voltage (*V*_oc_).^25^, ^31^, ^32^ In recent years, great progress has been made for the ternary OSCs, including new materials design, morphology optimization, and mechanism understanding.^30^, ^33^ Just recently, PCEs over 14% have been reported by Ding and co‐workers^22^ with a ternary device using a low band gap NFA acceptor, demonstrating great potential of ternary devices.

Recently, our^34^ and other groups^2^, ^35^ have almost simultaneously reported an A–D–A NFA **TTIC** with a broad and near infrared absorption ranging from 600 to 900 nm in the thin film. PCE of 10.87% was achieved when polymer **PBDB‐T** was used as the donor material in our case.^34^ In this work, based on the above molecule **TTIC**, we designed and synthesized a new NFA molecule **3TT‐FIC** (**Figure** **1**a) with three fused thieno[3,2‐b]thiophene as the central unit flanked with two electron‐withdrawing 2‐(5,6‐difluoro‐3‐oxo‐2‐2,3‐dihydro‐1H‐ioden‐1‐ylidene)malononitrile end groups. Considering the extended conjunction framework, we choose 4‐(2‐ethylhexylbenzene) as the side chain to enhance its solubility. As expected, **3TT‐FIC** showed maximum absorption peak located at 851 nm and a low optical bandgap of 1.25 eV. **PTB7‐Th**:**3TT‐FIC**‐based devices gave a PCE of 12.21% with a high *J*_sc_ of 25.89 mA cm^−2^. Considering the low EQE response in the range 300–500 nm for the above two‐component devices, we used phenyl‐C_71_‐butyric‐acid‐methyl ester (**PC_71_BM**) as the third component to fabricate the ternary device in order to lift the EQE response in the range of 300–500 nm and thus improve *J*_sc_ without sacrificing *V*_oc_ and FF. As expected, an evident EQE improvement was realized in the range 300–700 nm even with a small amount of 12% **PC_71_BM** in weight ratio. The PCE was elevated from 12.21% to 13.54% with evidently enhanced *J*_sc_ from 25.89 to 27.73 mA cm^−2^, and also slightly improved *V*_oc_ and FF. From the morphology analysis, the incorporation of small amount of **PC_71_BM** could also ameliorate the nanoscaled phase separation morphology, facilitate the charge transferring, and enhance the crystallinity in nonfullerene acceptor‐based devices.

**Figure 1:**
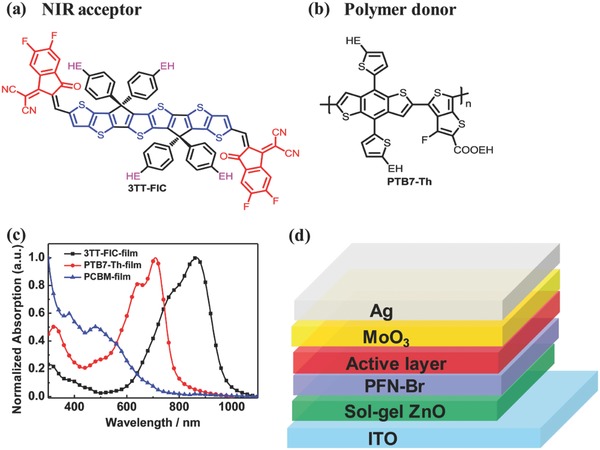
Chemical structures of a) **3TT‐FIC** nonfullerene acceptor and b) **PTB7‐Th** donor, c) UV–Vis absorption spectra of **PTB7‐Th**, **PC_71_BM**, and **3TT‐FIC** in neat film, d) the device structure.

The synthetic route of **3TT‐FIC** was shown in Figure S1 (Supporting Information), and the detailed synthesis procedure and the corresponding characterization data were given in the Supporting Information (SI). The precursor **1** was obtained by Stille coupling from two commercial materials and then nucleophilic addition of lithium reagent (4‐(2‐ethylhexylbenzene) lithium) with compound **1** to obtain the alcohol intermediate. The central unit **2** was synthesized using conc H_2_SO_4_ in tetrahydrofuran. The dialdehyde intermediate was prepared by the Vilsmeier–Haack reaction as a yellow‐red solid. Finally, the target molecule was obtained by Knoevenagel condensation in 86% yield. As shown in the thermogravimetric analysis (Figure S2, Supporting Information) measurement, **3TT‐FIC** demonstrated high thermal stability up to 340 °C without decomposition. The introduction of four 2‐ethylhexyl in the side chains made **3TT‐FIC** to have good solubility in chloroform, chlorobenzene, tetrahydrofuran, and other common solvents.

As shown in Figure S3 (Supporting Information), in dilute chloroform, **3TT‐FIC** shows broad and strong absorption with the λ_max_ located at 799 nm (ε = 2.1 × 10^5^ M^−1^ cm^−1^). In the neat film, **3TT‐FIC** exhibits broaden and red‐shifted absorption peaks located at 851 nm with the absorption edge extended to 995 nm, corresponding a low optical energy band gap (*E*_g_^opt^) of 1.25 eV (as shown in **Table** **1**). The combination of **3TT‐FIC** and **PTB7‐Th** shows broad and complementary absorption and provides a broad absorption ranging from 520 to 995 nm (Figure 1c). The density functional theory based on the B3LYP/6‐31G* bias set was used to evaluate the chemical geometry structure, in which the 2‐ethylhexyl side chains were replaced by methyl to simplify the calculation process. As revealed in Figure S4a (Supporting Information), the electron cloud density of highest occupied molecular orbital (HOMO) mainly located at the central building block, but its lowest unoccupied molecular orbital (LUMO) is delocalized preferably at the end group. From Figure S4b (Supporting Information), **3TT‐FIC** backbone shows preferable planarity with dihedral angle of 0^o^, which is beneficial for efficient π–π stacking and electron transfer. The HOMO/LUMO energy levels of **3TT‐FIC** were determined by cyclic voltammetry measurement using solid film in its acetonitrile solution (Figure S5a, Supporting Information). The HOMO and LUMO of **3TT‐FIC** were calculated to be −5.42 and −4.17 eV (*E*_HOMO_ + *E*_g_^opt^), respectively.

**Table 1:** Photophysical and electrochemical parameters of **3TT‐FIC**

Comp.	λ_max_^sol^ [nm]	λ_max_^film^ [nm]	λ_onset_^film^ [nm]a)	*E*_g_^opt^ [eV]a)	HOMO [eV]	LUMO [eV]	ε_max_ (M^−1^ cm^−1^)a)
**3TT‐FIC**	799	851	995	1.25	−5.42	−4.17	2.1 × 10^5^

^a)^ *E*_g_^opt^ = 1240/λ_onset_ (eV).

The OSCs devices were fabricated using an inverted structure of ITO/ZnO/PFN‐Br/active layers/MoO_3_/Ag, where the active layers contained the binary blend of **PTB7‐Th**: **3TT‐FIC** or ternary blend of **PTB7‐Th**:**3TT‐FIC**: **PC_71_BM**. The detailed device optimization procedures were recorded in the supporting information (Tables S1 and S2, Supporting Information). For the binary devices, the optimal weight ratio of **PTB7‐Th**: **3TT‐FIC** was 1:1.2 (with 10 mg **PTB7‐Th** and 12 mg **3TT‐FIC** dissolved in chlorobenzene) with the active layer thickness of ≈100 nm. A high PCE of 12.21% with *J*_sc_ of 25.89 mA cm^−2^, *V*_oc_ of 0.662 V and a fill factor (FF) of 0.71 was achieved via a solvent vapor annealing process with chloroform. The current density–voltage (*J–V*) curves of the optimized device are shown in **Figure** **2**a, and the corresponding photovoltaic parameters are summarized in **Table** **2**. As shown in Figure 2b, the binary OSCs gave a broad EQE response in the range from 300 to 1000 nm with high EQE values around 0.80 in the range from 660 to 820 nm. The calculated integrated *J*_sc_ value from EQE curve was 24.64 mA cm^−2^, which is consistent with the measured value (25.89 mA cm^−2^) with a mismatch of 4.8%.

**Figure 2:**
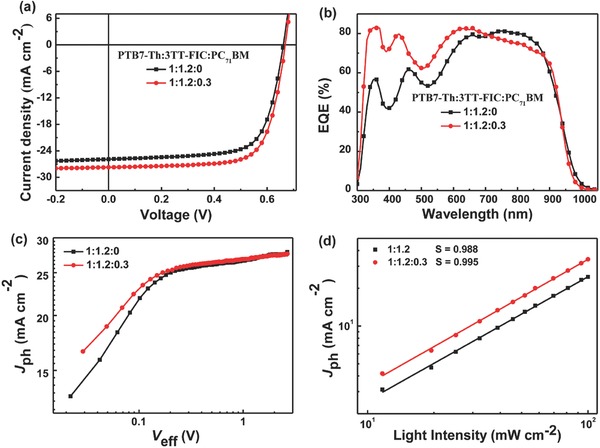
a) Current density–voltage (*J*–*V*) curves of **PTB7‐Th**:**3TT‐FIC** based binary device and **PTB7‐Th**:**3TT‐FIC**:**PC_71_BM** based ternary device under one sun illumination (AM 1.5 G 100 mW cm^−2^). b) EQE spectra of the corresponding devices. c) *J*_ph_ versus *V*_eff_ and d) light‐intensity (*P*) dependence of *J*_sc_ measurement of the devices.

**Table 2:** The photovoltaic data of the optimized devices based on **PTB7‐Th**:**3TT‐FIC** and **PTB7‐Th**:**3TT‐FIC**:**PC_71_BM** under the illumination of AM 1.5G (100 mW cm^−2^)

Comp.	*V*_oc_ [V]	FF	*J*_sc_ *^J–V^* [mA cm^−2^]	PCE [%]a)
1:1.2:0	0.662	0.712	25.89	12.21 (11.96 ± 0.25)
1:1.2:0.15	0.666	0.719	27.36	13.10 (12.79 ± 0.31)
1:1.2:0.30	0.669	0.730	27.73	13.54 (13.33 ± 0.21)
1:1.2:0.45	0.671	0.690	27.29	12.63 (12.28 ± 0.35)

^a)^ The PCE values were calculated from 20 devices for each case.

Note from Figure 2b, the EQE response in the short wavelength range is clearly lower than that in the longer wavelength range. Although with overall weak absorption, **PC_71_BM** has relatively good absorption and could enhance EQE response in the short wavelength range in both binary and ternary devices.^28^, ^29^, ^32^ Also for the ternary devices, **PC_71_B**M could form cascade energy level to facilitate the charge separation and tune the active layer morphology.^25^, ^32^, ^36^ Thus, in order to lift the short wavelength range EQE and thus improve the *J*_sc_, we chose **PC_71_BM** as the third component to fabricate the **PTB7‐Th**: **3TT‐FIC**:**PC_71_BM** ternary blend device. After carefully device optimizations, the best weight ratio of **PTB7‐Th**: **3TT‐FIC**: **PC_71_BM** was 1:1.2:0.3 in wt% (with 3 mg **PC_71_BM** added into the binary device), and a high PCE of 13.54% with an enhanced *J*_sc_ of 27.73 mA cm^−2^, FF of 0.73 and *V*_oc_ of 0.669 V was achieved. In comparison with the binary device, the ternary OSCs showed an evidently enhanced EQE response from 300 to 700 nm after incorporation of **PC_71_BM**. Although the EQE response in the long wavelength range 750–900 nm was slightly downshifted as expected, the calculated integrated *J*_sc_ in the whole EQE response window of the ternary device was improved to 26.41 mA cm^−2^ (mismatch factor of 4.7% compared with *J*_sc_ from *J–V* measurement), compared with that of 25.89 mA cm^−2^ for the binary device.

The photocurrent (*J*_ph_) versus the effective applied voltage (*V*_eff_) (Figure 2c) measurements were conducted to investigate and compare the exciton dissociation and charge collection dynamics for the binary and ternary devices. The *J*_ph_ was calculated from the value of *J*_L_−*J*_D_, where *J*_L_ and *J*_D_ represent the current density under illumination and in the dark. The *V*_eff_ is determined by the *V*_0_−*V*_a_, where *V*_0_ is the voltage when *J*_L_ = *J*_D_ and *V*_a_ is the applied voltage.^37^, ^38^ As depicted in **Figure** **3**, when *V*_eff_ exceeds 1.6 V, *J*_ph_ becomes saturated (*J*_sat_), indicating both types of the devices derived high charge extraction probability under high voltages. The ratio of *J*_ph_/*J*_sat_ calculated from the ternary device was 0.96, which is higher than that of the binary device (0.94) under short circuit conditions. In addition, under maximal power output conditions, the ternary devices also gave a higher value (0.85) of *J*_ph_/*J*_sat_ than that (0.83) of the binary devices. These results indicate that the addition of **PC_71_BM** in the **PTB7‐Th**:**3TT‐FIC** based device offers higher exciton dissociation and more efficient charge collection efficiency. The light‐intensity dependence of *J*_sc_ results are shown in Figure 2d. According to the equation of *J*_sc_∝*P* ^α^,^37^ a slope of 0.995 and 0.988 were obtained for the ternary and binary based devices, respectively, indicating that both the ternary and binary devices showed low bimolecular recommendation with the slope value close to 1. The space charge limit current measurement was used to measure the charge mobility and the corresponding curves are listed in Figure S6 a,b (Supporting Information). The electron and hole mobility of the ternary device are 2.28 × 10^−4^ and 2.16 × 10^−4^ cm^2^ V^−1^ s^−1^, respectively, which are slightly larger and more balanced than those of the binary device (1.67 × 10^−4^ and 2.09 × 10^−4^ cm^2^ V^−1^ s^−1^). These results are consistent with the improved FFs of the ternary devices.

**Figure 3:**
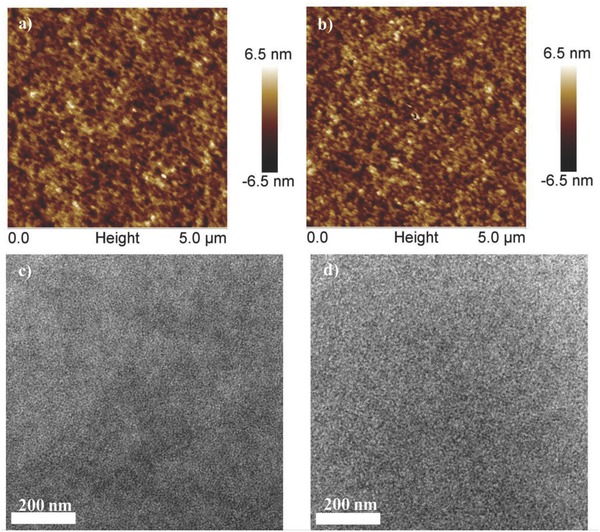
AFM images for a) **PTB7‐Th**:**3TT‐FIC** blend film and b) **PTB7‐Th**:**3TT‐FIC**:**PC71BM** blend film. TEM images for c) **TB7‐Th**:**3TT‐FIC** blend film and d) **PTB7‐Th**:**3TT‐FIC**:**PCBM** blend film.

The atomic force microscopy (AFM) under typing‐mode and transmission electron microscopy (TEM) were utilized to study the morphologies of the **PTB7‐Th**:**3TT‐FIC** blend film and also the corresponding ternary film. As shown in AFM images (Figure 3a,b), the morphology of the binary and ternary blend films showed little difference with nearly the same root‐mean‐square surface roughness values of 1.60 and 1.59 nm. In TEM images (Figure 3c,d), both the binary and ternary blend film showed good nanoscale fibrillar structure phase separation, with the ternary based film showing relatively clearer and better continuous phase separation. The 2D grazing incidence wide‐angle X‐ray diffraction (GIXD) was measured to investigate the microstructural molecular packing in the blend of **PTB7‐Th**:**3TT‐FIC** and **PTB7‐Th**:**3TT‐FIC**:**PC_71_BM** films (**Figure** **4**a,b). The single component GIXD results are shown in Figure S7 (Supporting Information), where **PTB7‐Th** polymer shows a dominant face‐on orientation with a (100) reflection of 0.27 Å^−1^ at the in‐plane (IP) direction and a sharp (010) reflection at the out‐of‐plane (OOP) direction of 1.58 Å^−1^. The **3TT‐FIC** acceptor shows a wide (100) reflection at 0.35 Å^−1^ and a π–π stacking in the OOP direction at 1.80 Å^−1^. The diffraction peak of **PC_71_BM** located at 1.32 Å^−1^ both in the IP and OOP direction.^24^, ^25^ However, there was no evident **PC_71_BM** diffraction peak in the ternary blend film owing to the minor addition of **PC_71_BM**. From the line‐cut profiles (Figure 4c), the binary and ternary blend films exhibit relatively weak (100) diffraction peaks in both the in‐plane and out‐of‐plane directions. The binary blend film has strong (010) diffraction peaks in the out‐of‐plane direction, due to the combined diffraction features of **PTB7‐Th** and **3TT‐FIC** and located at 1.66 Å^−1^ and 1.81 Å^−1^ by Gaussian fitting with the full‐width at half‐maximum (Δq) 0.40 and 0.23 Å, respectively (Figure S8a, Supporting Information).^33^ From Scherrer equation,^39^ the crystal coherence length (CCL) in the (010) diffraction direction was estimated to be 24.6 Å for **3TT‐FIC** with a π–π stacking distance of 3.47 Å. In the ternary film, contrary to the binary blend film, the (010) peak of **3TT‐FIC** was located at 1.83 Å^−1^ with a Δq of 0.16 Å, which corresponds with an enhanced CCL of 34.4 Å and a shorter π–π stacking distance of 3.43 Å for **3TT‐FIC** (Figure S8b, Supporting Information). For the **PTB7‐Th,** in the ternary blend film, the (010) direction located at 1.67^−1^ Å with a Δq of 0.40 Å, which represents a similar (010) diffraction peak and crystallinity with the binary blend film. These results indicate that the incorporation of **PC_71_BM** have increased the crystallinity of **3TT‐FIC** and its π–π stacking pattern in the ternary device. This is consistent with the measured charge mobility and the FF values discussed above.

**Figure 4:**
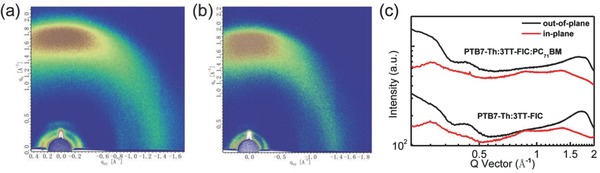
2D GIXD images of the a) binary and b) ternary blend films. c) The out‐of‐plane (black line) and in‐plane (red line) line‐cut profiles.

In summary, a new NFA named **3TT‐FIC** has been designed and synthesized with an extending conjugated central core and electron‐withdrawing difluoro substituted indanone end group. **3TT‐FIC** possessed strong and broad absorption with the maximum absorption peak located at 995 nm and a low optical bandgap of 1.25 eV in the thin film. The binary device based on **3TT‐FIC** and **PTB7‐Th** gave a power conversion efficiency of 12.21% with a high *J*_sc_ of 25.89 mA cm^−2^. From the perspective of complementary absorption and EQE response improvement, a ternary device based on the above binary OSC by introducing **PC_71_BM** as the third component was fabricated and studied. A high PCE with value of 13.54% and *J*_sc_ of 27.73 mA cm^−2^ was achieved for the ternary device. The PCE enhancement was mainly ascribed to the improved EQE response in the visible range and thus *J*_sc_ after the addition of **PC_71_BM**. Meanwhile, the *V*_oc_ nearly kept unchanged and FF was also slightly improved owing to the more balanced hole and electron mobilities and better morphology with the introduction of **PC_71_BM**. This work demonstrates that when the additional component was chosen properly, higher performance could be further obtained above the corresponding optimized binary devices.

## Conflict of Interest

The authors declare no conflict of interest.

## Supporting information

Supplementary

## References

[1] F. Liu , Z. Zhou , C. Zhang , J. Zhang , Q. Hu , T. Vergote , F. Liu , T. P. Russell , X. Zhu , Adv. Mater. 2017, 29, 1606574. 10.1002/adma.201606574 28323352

[2] W. Wang , C. Yan , T. K. Lau , J. Wang , K. Liu , Y. Fan , X. Lu , X. Zhan , Adv. Mater. 2017, 29, 1701308. 10.1002/adma.201701308 28608531

[3] Y. Li , G. Xu , C. Cui , Y. Li , Adv. Energy Mater. 2017, 8, 1701791.

[4] M. Kaltenbrunner , M. S. White , E. D. Glowacki , T. Sekitani , T. Someya , N. S. Sariciftci , S. Bauer , Nat. Commun. 2012, 3, 770. 22473014 10.1038/ncomms1772 PMC3337988

[5] T. Kim , J. H. Kim , T. E. Kang , C. Lee , H. Kang , M. Shin , C. Wang , B. Ma , U. Jeong , T. S. Kim , B. J. Kim , Nat. Commun. 2015, 6, 8547. 26449658 10.1038/ncomms9547 PMC4633811

[6] Z. Zhang , Z. Yang , J. Deng , Y. Zhang , G. Guan , H. Peng , Small 2015, 11, 675. 25236579 10.1002/smll.201400874

[7] W. Zhao , S. Li , H. Yao , S. Zhang , Y. Zhang , B. Yang , J. Hou , J. Am. Chem. Soc. 2017, 139, 7148. 28513158 10.1021/jacs.7b02677

[8] Z. Xiao , X. Jia , D. Li , S. Wang , X. Geng , F. Liu , J. Chen , S. Yang , T. P. Russell , L. Ding , Sci. Bull. 2017, 62, 1494. 10.1016/j.scib.2017.10.017 36659424

[9] J. Zhu , Z. Ke , Q. Zhang , J. Wang , S. Dai , Y. Wu , Y. Xu , Y. Lin , W. Ma , W. You , X. Zhan , Adv. Mater. 2017, 30, 1704713. 10.1002/adma.201704713 29168900

[10] S. J. Xu , Z. Zhou , W. Liu , Z. Zhang , F. Liu , H. Yan , X. Zhu , Adv. Mater. 2017, 29, 1704510. 10.1002/adma.201704510 28985002

[11] B. Kan , J. Zhang , F. Liu , X. Wan , C. Li , X. Ke , Y. Wang , H. Feng , Y. Zhang , G. Long , R. H. Friend , A. A. Bakulin , Y. Chen , Adv. Mater. 2017, 30, 1704904. 10.1002/adma.201704904 29205535

[12] L. Lu , T. Zheng , Q. Wu , A. M. Schneider , D. Zhao , L. Yu , Chem. Rev. 2015, 115, 12666. 26252903 10.1021/acs.chemrev.5b00098

[13] O. Ostroverkhova , Chem. Rev. 2016, 116, 13279. 27723323 10.1021/acs.chemrev.6b00127

[14] Y. Jin , Z. Chen , S. Dong , N. Zheng , L. Ying , X. F. Jiang , F. Liu , F. Huang , Y. Cao , Adv. Mater. 2016, 28, 9811. 27647512 10.1002/adma.201603178

[15] Y. Cui , C. Yang , H. Yao , J. Zhu , Y. Wang , G. Jia , F. Gao , J. Hou , Adv. Mater. 2017, 29, 1703080. 10.1002/adma.201703080 28977709

[16] Y. Lin , J. Wang , Z. G. Zhang , H. Bai , Y. Li , D. Zhu , X. Zhan , Adv. Mater. 2015, 27, 1170. 25580826 10.1002/adma.201404317

[17] F. Zhao , S. Dai , Y. Wu , Q. Zhang , J. Wang , L. Jiang , Q. Ling , Z. Wei , W. Ma , W. You , C. Wang , X. Zhan , Adv. Mater. 2017, 29, 1700144. 10.1002/adma.201700144 28295734

[18] Y. Cui , H. Yao , B. Gao , Y. Qin , S. Zhang , B. Yang , C. He , B. Xu , J. Hou , J. Am. Chem. Soc. 2017, 139, 7302. 28497691 10.1021/jacs.7b01493

[19] B. Chen , X. Zheng , Y. Bai , N. P. Padture , J. Huang , Adv. Energy Mater. 2017, 7, 1602400.

[20] L. Zuo , J. Yu , X. Shi , F. Lin , W. Tang , A. K. Jen , Adv. Mater. 2017, 29, 1702547. 10.1002/adma.201702547 28692752

[21] H. Lu , X. Xu , Z. Bo , Sci. China Mater. 2016, 59, 444.

[22] Z. Xiao , X. Jia , L. Ding , Sci. Bull. 2017, 62, 1562. 10.1016/j.scib.2017.11.003 36659472

[23] L. Lu , W. Chen , T. Xu , L. Yu , Nat. Commun. 2015, 6, 7327. 26041586 10.1038/ncomms8327 PMC4468850

[24] G. Zhang , K. Zhang , Q. Yin , X.‐F. Jiang , Z. Wang , J. Xin , W. Ma , H. Yan , F. Huang , Y. Cao , J. Am. Chem. Soc. 2017, 139, 2387. 28127955 10.1021/jacs.6b11991

[25] H. Lu , J. Zhang , J. Chen , Q. Liu , X. Gong , S. Feng , X. Xu , W. Ma , Z. Bo , Adv. Mater. 2016, 28, 9559. 27620971 10.1002/adma.201603588

[26] T. Liu , Y. Guo , Y. Yi , L. Huo , X. Xue , X. Sun , H. Fu , W. Xiong , D. Meng , Z. Wang , F. Liu , T. P. Russell , Y. Sun , Adv. Mater. 2016, 28, 10008. 27717048 10.1002/adma.201602570

[27] S. L. Chang , F. Y. Cao , W. C. Huang , P. K. Huang , C. S. Hsu , Y. J. Cheng , ACS Appl. Mater. Interfaces 2017, 9, 24797. 28660755 10.1021/acsami.7b06650

[28] T. H. Lee , M. A. Uddin , C. Zhong , S.‐J. Ko , B. Walker , T. Kim , Y. J. Yoon , S. Y. Park , A. J. Heeger , H. Y. Woo , J. Y. Kim , Adv. Energy Mater. 2016, 6, 1600637.

[29] T. Liu , X. Xue , L. Huo , X. Sun , Q. An , F. Zhang , T. P. Russell , F. Liu , Y. Sun , Chem. Mater. 2017, 29, 2914.

[30] X. Du , X. Jiao , S. Rechberger , J. D. Perea , M. Meyer , N. Kazerouni , E. Spiecker , H. Ade , C. J. Brabec , R. H. Fink , T. Ameri , Macromolecules 2017, 50, 2415.

[31] X. Xu , Z. Bi , W. Ma , Z. Wang , W. C. H. Choy , W. Wu , G. Zhang , Y. Li , Q. Peng , Adv. Mater. 2017, 29, 1704271. 10.1002/adma.201704271 29044740

[32] Y. Zhang , D. Deng , K. Lu , J. Zhang , B. Xia , Y. Zhao , J. Fang , Z. Wei , Adv. Mater. 2015, 27, 1071. 25655181 10.1002/adma.201404902

[33] J. Zhang , Y. Zhang , J. Fang , K. Lu , Z. Wang , W. Ma , Z. Wei , J. Am. Chem. Soc. 2015, 137, 8176. 26052738 10.1021/jacs.5b03449

[34] H.‐H. Gao , Y. Sun , X. Wan , B. Kan , X. Ke , H. Zhang , C. Li , Y. Chen , Sci. China Mater. 2017, 60, 819.

[35] X. Shi , L. Zuo , S. B. Jo , K. Gao , F. Lin , F. Liu , A. K. Y. Jen , Chem. Mater. 2017, 29, 8369.

[36] M. Zhang , J. Wang , F. Zhang , Y. Mi , Q. An , W. Wang , X. Ma , J. Zhang , X. Liu , Nano Energy 2017, 39, 571.

[37] C. M. Proctor , M. Kuik , T.‐Q. Nguyen , Prog. Polym. Sci. 2013, 38, 1941.

[38] P. W. M. Blom , V. D. Mihailetchi , L. J. A. Koster , D. E. Markov , Adv. Mater. 2007, 19, 1551.

[39] D.‐M. Smilgies , J. Appl. Crystallogr. 2009, 42, 1030. 19953189 10.1107/S0021889809040126 PMC2779741

