# Supplementary material for: A New Nonfullerene Acceptor with Near Infrared Absorption for High Performance Ternary‐Blend Organic Solar Cells with Efficiency over 13%
Source: Adv Sci (Weinh). 2018 Mar 25;5(6):1800307. doi: 10.1002/advs.201800307 (PMC6010751; doi:10.1002/advs.201800307)
Supplement: Supplementary file 1 — Supplementary [file ADVS-5-1800307-s001.pdf]

Copyright WILEY-VCH Verlag GmbH & Co. KGaA, 69469 Weinheim, Germany, 2018.

ADVANCED SCIENCE
Open Access

Supporting Information

for *Adv. Sci.,* DOI: 10.1002/advs.201800307

A New Nonfullerene Acceptor with Near Infrared Absorption for High Performance Ternary-Blend Organic Solar Cells with Efficiency over 13%

Huan-Huan Gao, Yanna Sun, Xiangjian Wan, Xin Ke, Huanran Feng, Bin Kan, Yanbo Wang, Yamin Zhang, Chenxi Li, and Yongsheng Chen**

Copyright WILEY-VCH Verlag GmbH & Co. KGaA, 69469 Weinheim, Germany, 2016.

Supporting Information

A new Non-Fullerene Acceptor with Near Infrared Absorption for High Performance Ternary-Blend Organic Solar Cells with Efficiency over 13%

Huan-huan Gao, Yanna Sun, Xiangjian Wan*, Xin Ke, Huanran Feng, Bin Kan, Yanbo Wang, Yamin Zhang, Chenxi Li, Yongsheng Chen*

Experiment Section

1.Materials

All the chemicals were purchased from commercial business and without further purification and the solvents were treated according to the standard procedures. **PTB7-Th** was purchased from One Materials.

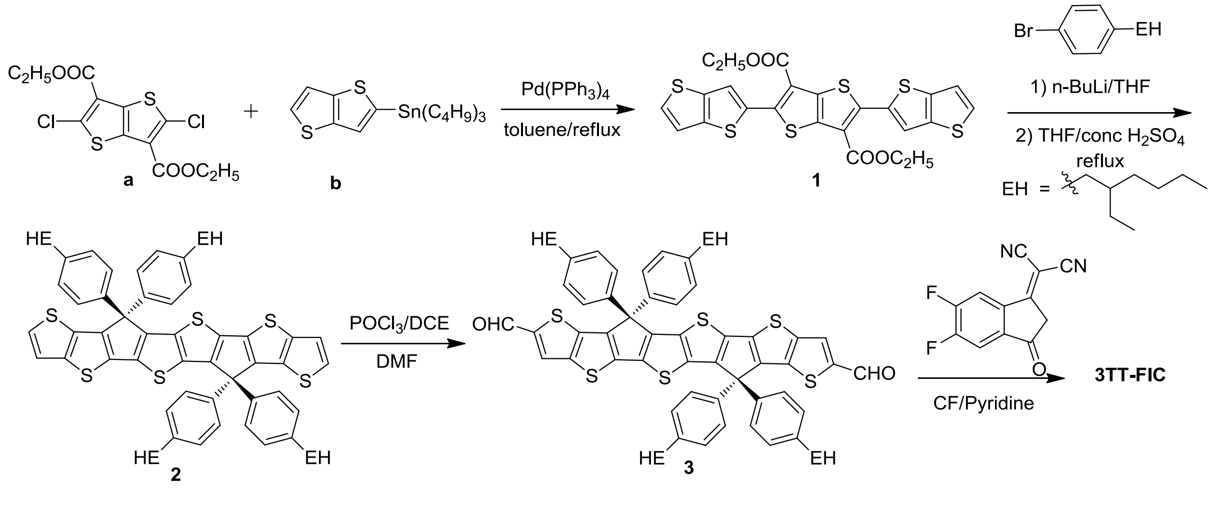

Figure S1. Synthetic routes of **3TT-FIC.**

1: Under the atmosphere of argon, the ingredient **a**, 1 g (2.83 mmol), **b**, 3.644 g (8.49 mmol), and $Ph(PPh_3)_4$, 164 mg (0.142 mmol) were added into a 100 mL two neck round-bottom flask and degassed with argon three times. Then 30 mL anhydrous toluene was injected into the reaction system and stirred at 110 ℃ overnight. The reaction solution was cooled to room temperature and poured into 100 mL anhydrous methanol, then filtered and washed with a great deal of petroleum ether, ethyl acetate and methanol. Due to the poor solubility the raw product were used to the next reaction without further purification. Due to the poor solubility, we do not obtain the NMR data.

2: Under the atmosphere of argon, 2-ethylhexylbenzene, 1.2 g (4.46 mmol), in anhydrous THF (30 mL) and then 3 mL n-BuLi (1.6 M) was added into the reaction system dropwise in -78 °CThe reaction was stirred at -78 ℃ for 1 h. Compound **1**, 500 mg (0.89 mmol) was added into the system quickly, and the reaction was stirred at room temperature for another 12 h. The reagent solution was washed with brine for three times and dried over anhydrous Na_2SO_4. The solvent was removed under vacuum. The crude product was dissolved in tetrahydrofuran 30 mL and 0.3 mL conc H_2SO_4 was added as the catalyst. The reaction was stirred at 90 ℃ for 2 h and quenched with 10 mL ice water and washed with water for 4 times. The solvent was removed under vacuum, and the crude product was purified by column chromatography using CH_2Cl_2/PE (1:40) as the eluent to give compound **2** as a yellow-red solid (62%). ^1H NMR (400 MHz, $CDCl_3$) δ 7.25 – 7.22 (m, 2H), 7.15 (d, *J* = 8.1 Hz, 8H), 7.07 (d, *J* = 8.2 Hz, 8H), 2.47 (d, *J* = 6.9 Hz, 8H), 1.54 – 1.49 (m, 4H), 1.26 – 1.21 (m, 32H), 0.86 – 0.81 (m, 24H). HR-MS (MALDI): m/z $[M]^+$ calcd for $C_{76}H_{88}S_6$, 1192.5210, found, 1192.5210.

3: Under the atmosphere of argon, anhydrous N,N-dimethylformamide (5 mL) was added. Then anhydrous phosphorus oxychloride ($POCl_3$) (400 μL) was injected into the reaction system drop by drop under the ice-water bath. The reaction was stirred under 0 ℃ for another 30 minutes. After

that, the ice-water bath was removed and stirred in the room temperature for 3 hours to gain the Vilsmerier reagent. A mixture of **2**, 500 mg (0.42 mmol) in 1,2-dichloroethane 80 mL and the reaction was degassed with argon for 15 minutes and then the Vilsmerier reagent was added into the reaction slowly and stirred at room atmosphere for another 1 h. finally the reaction was stirred at 85 ℃ for another 12 hours. 30 mL saturated sodium acetate solution was added slowly to quench the reaction. The reagent solution was washed with water for three times and dried over anhydrous Na_2SO_4. The solvent was removed under vacuum, and the crude product was purified by column chromatography using CH_2Cl_2/PE (1:1) as the eluent to give compound **3** as a red solid (90%). ^{1}H NMR (400 MHz, $CDCl_3$) δ 9.87 (s, 2H), 7.92 (s, 2H), 7.09 (d, *J* = 4.7 Hz, 16H), 2.47 (d, *J* = 7.0 Hz, 8H), 1.55 – 1.50 (m, 4H), 1.26 – 1.19 (m, 32H), 0.86 – 0.81 (m, 24H). ^{13}C NMR (101 MHz, $CDCl_3$) δ 182.65, 150.69, 147.27, 145.55, 143.43, 141.65, 140.35, 138.25, 137.14, 136.46, 129.80, 127.54, 62.40, 40.82, 40.80, 39.74, 32.31, 28.83, 28.79, 25.50, 22.95, 14.09, 10.76, 10.74. HR-MS (MALDI): m/z $[M]^+$ calcd for $C_{78}H_{88}O_2S_6$, 1248.5109, found, 1248.5229.

Synthesis of 3TT-FIC. A mixture of compound **3**, 100 mg (0.08 mmol), 3-(dicyanomethylidene)-indan-1-one 92 mg (0.4 mmol) in chloroform 20 mL under argon atmosphere. The reaction was stirred at room temperature for 12 hours. The organic phase was washed with water for three times and dried over anhydrous Na_2SO_4. The solvent was removed under vacuum, and the crude product was purified by column chromatography using CF/PE (1:1) as the eluent to give the crude product was purified with further recrystalization using CF/methanol to obtain compound **3TT-FIC** as a green solid (88%). ^{1}H NMR (400 MHz, $CDCl_3$) δ 8.83 (s, 2H), 8.52 (dd, *J* = 9.7, 6.6 Hz, 2H), 8.19 (s, 2H), 7.66 (t, *J* = 7.4 Hz, 2H), 7.18 (d, *J* = 8.2 Hz, 8H), 7.14 (d, *J* = 8.2 Hz, 8H), 2.49 (d, *J* = 6.8 Hz, 8H), 1.56 – 1.51 (m, 4H), 1.27 – 1.20 (m, 32H), 0.87 – 0.80 (m, 24H). ^{13}C NMR (101 MHz, $CDCl_3$) δ 185.84, 158.22, 155.76, 155.62, 152.98, 142.02, 138.06, 138.00, 136.61, 134.47, 134.41, 130.01, 127.54, 115.03, 114.81, 114.41, 114.34, 112.64, 112.46, 69.16, 62.55, 40.79, 39.75, 32.31, 29.34, 28.80, 27.22, 25.51, 22.97, 14.10, 10.75. HR-MS (MALDI): m/z $[M]^+$ calcd for $C_{102}H_{92}F_4N_4O_2S_6$, 1672.5481, found, 1672.5516.

2. Fabrication of OSCs

The OSCs devices were fabricated using an inverted structure of ITO/ZnO/PFN-Br/active layers/MoO_3/Ag. The indium tin oxide (ITO)-coated glass substrates were cleaned by ultrasonic treatment in detergent, deionized water, acetone, and isopropyl alcohol under ultrasonication for 15 min each and subsequently dried by a nitrogen blow. Subsequently, a 30 nm thick layer of ZnO was deposited by spin-coating a ZnO precursor solution on the top of the ITO glass substrates at 3000 rpm for 40 s. After being baked at 200 °C in air for 60 min, the ZnO-coated substrates were transferred into a nitrogen-filled glove box. ZnO film thickness is ~30 nm. In order to fun-tune the interfacial properties a thin film of PFN-Br was spin-coated on ZnO. Subsequently, the binary blend of **PTB7-Th**:**3TT-FIC** or ternary blend of **PTB7-Th**:**3TT-FIC**:**PC71BM** in chlorobenzene (CB) with DIO additive was spin-coated onto ZnO layer. MoO_3 (~10 nm) and Ag (~80 nm) was successively evaporated onto the active layer through a shadow mask (pressure ca. 10^{-4} Pa). The effective area for the devices is 4 mm^2.

3. Characterization and measurement

The ^1H and ^{13}C nuclear magnetic resonance (NMR) spectra were taken on a Bruker AV400 Spectrometer. The HR-MS data were recorded on Varian 7.0T FT-MS. The thermogravimetric analysis (TGA) was carried out on a NETZSCH STA 409PC instrument under purified nitrogen gas flow. The heating rate for TGA testing is 15 °C min^{-1}, UV–Vis spectra were obtained with a JASCO V-570 spec-trophotometer. Cyclic voltammetry (CV) experiments were performed with a LK98B II microcomputer-based Electro-chemical Analyzer. All CV measurements were carried out at room temperature with a conventional three-electrode configuration employing a glassy carbon electrode as the working electrode, a saturated calomel electrode (SCE) as the reference electrode, and a Pt wire as the counter electrode. Acetonitrile was distilled from calcium hydride under dry nitrogen immediately prior to use. Tetrabutylammonium phosphorus hexafluoride (Bu_4NPF_6, 0.1 M) in acetonitrile was used as the supporting electrolyte; the scan rate was 100 mV s^{-1}. Atomic force microscope (AFM) was performed using MultiMode 8 atomic force microscope in tapping mode.

SCLC mobility was measured using a diode configuration of ITO/ PEDOT:PSS/ **PTB7-Th**:acceptor/ Au for hole and Al/ **PTB7-Th**: acceptor / Al for electron by taking the dark current density in the range of 0-2 V and fitting the results to a space charge limited form, where SCLC is described by:

$$J = \frac{9\varepsilon_0 \varepsilon_r \mu_0 V^2}{8L^3}$$

where J is the current density, L is the film thickness of the active layer, μ_0 is the hole or electron mobility, ε_r is the relative dielectric constant of the transport medium, ε_0 is the permittivity of free space (8.85×10^{-12} F m^{-1}), V (= V_{appl} - V_{bi}) is the internal voltage in the device, where V_{appl} is the applied voltage to the device and V_{bi} is the built-in voltage due to the relative work function difference of the two electrodes. The current density-voltage (*J*–*V*) curves of photovoltaic devices were obtained by a Keithley 2400 source-measure unit. The photocurrent was measured under illumination simulated 100 mW cm^{-2} AM 1.5G irradiation using a xen-on-lamp-based solar simulator [SAN-EI XES-70S1 (AM 1.5G)] in an argon filled glove box. External quantum efficiencies (EQE) were measured using Stanford Re-search Systems SR810 lock-in amplifier.

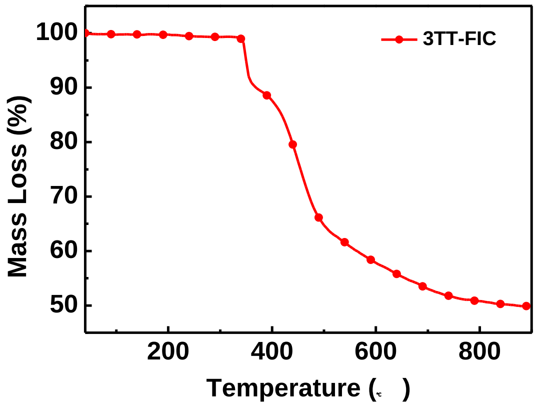

Figure S2. The thermogravimetric analysis (TG) plots of 3**TT-FIC** with a heating rate of 15 °C/min under nitrogen.

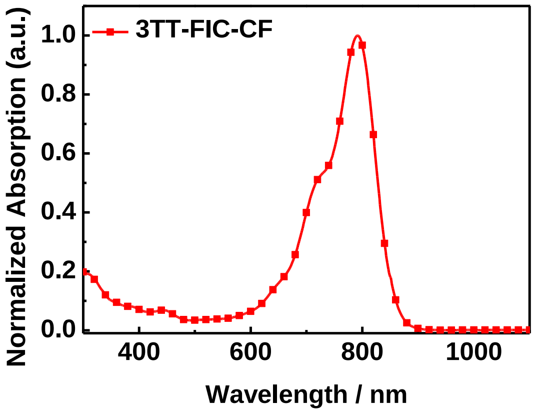

Figure S3. UV-Vis absorption of **3TT-FIC** in dilute chloroform.

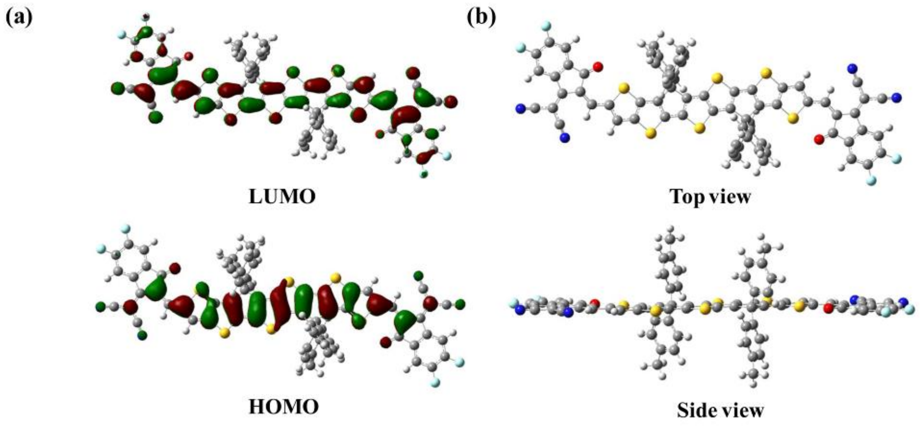

Figure S4. The calculated electron distribution of the molecular frontier orbitals and optimized geometries of **3TT-FIC** using DFT calculations[1].

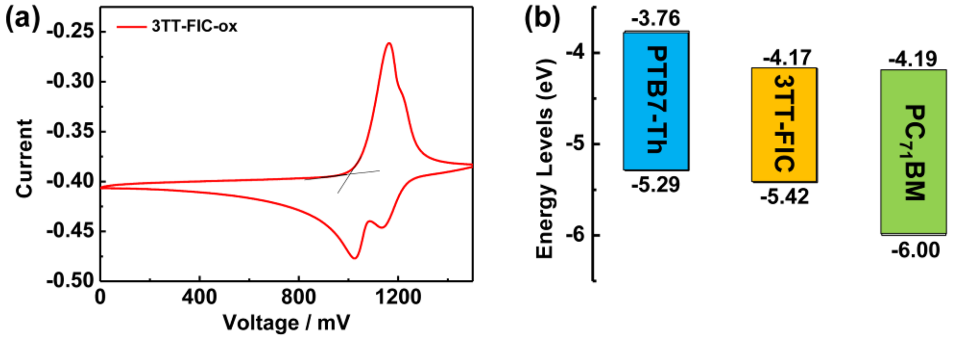

Figure S5. Cyclic voltammetry (CV) plot of **3TT-FIC** (a) in the solid film and the energy levels of **PTB7-Th**, **3TT-FIC** and **PC$_{71}$BM**.

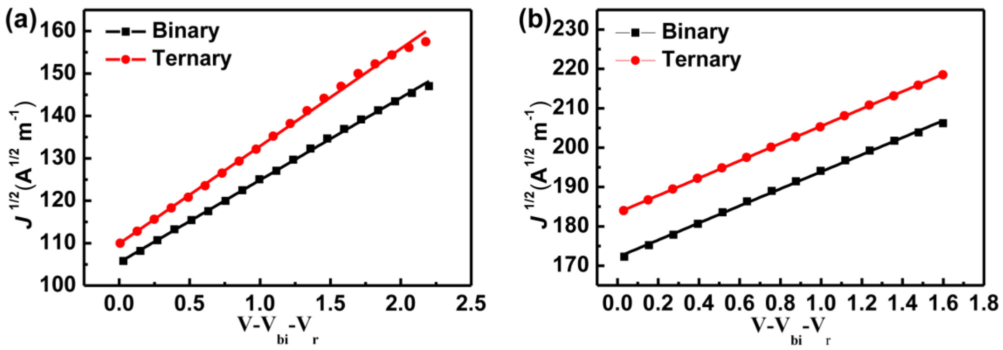

Figure S6. The electron (a) and hole (b) mobility based on the binary and ternary OSCs devices.

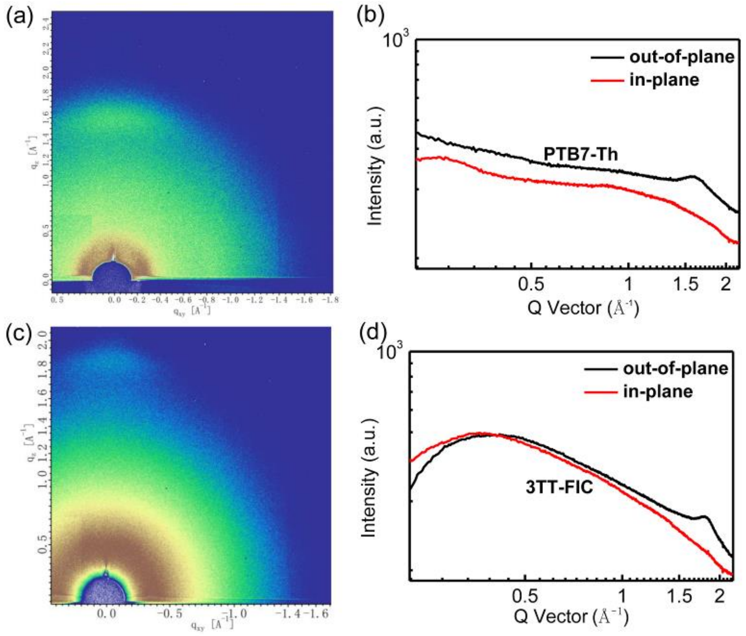

Figure S7. 2D GIXD imsges of **PTB7-Th** (a) and **3TT-FIC** (c) neat films. The corresponding out-of-plane (black line) and in-plane (red line) line-cut profiles (b) and (d).

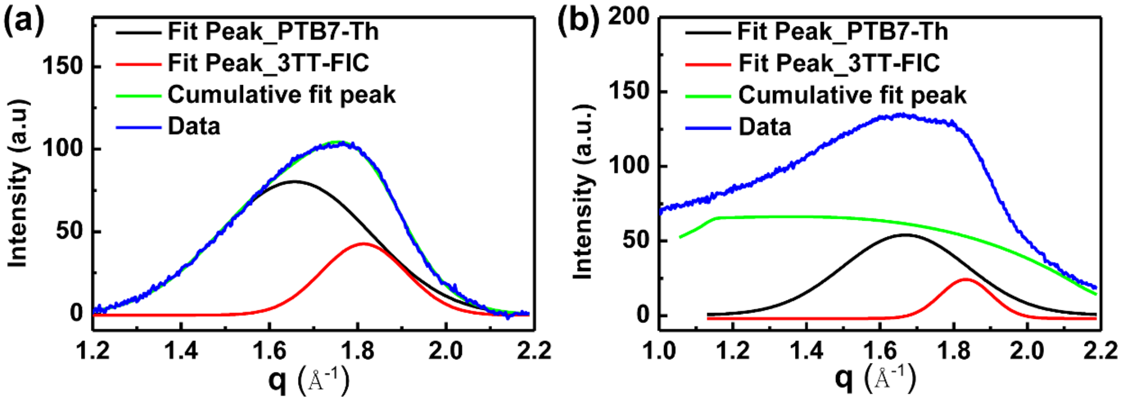

Figure S8. The Gaussian fitting for out-of-plane cut for (a) the binary blend film and (b) the ternary blend film.

Table S1. Photovoltaic performances based on **PTB7-Th**:**3TT-FIC**:**PCBM** devices with different active layer thickness under one sun illumination (AM 1.5 G, 100 mW cm^{-2}).

Comp.	λ_{max}^{film} [nm]	λ_{onset}^{film} [nm]$^{a)}$	E_g^{opt} [eV]	HOMO [eV]	LUMO [eV]
**PTB7-Th**	709	812	1.53	-5.29	-3.76
**PC$_{71}$BM**	480	685	1.81	-6.00	-4.19

a) E_g^{opt}=1240/λ_{edge} (eV).

Table S2. Photovoltaic performances based on **PTB7-Th**:**3TT-FIC**:**PC$_{71}$BM** devices with different active layer thickness under one sun illumination (AM 1.5 G, 100 mW cm^{-2}).

Thickness [nm]	V_{oc} [V]	FF	J_{sc}^{CV} [mA cm^{-2}]	PCE [%]$^{a)}$
116	0.667	0.724	27.45	13.26 (13.02 ± 0.24)
102	0.669	0.730	27.73	13.54 (13.33 ± 0.21)
90	0.668	0.718	26.61	12.76 (12.46 ± 0.30)
77	0.672	0.722	25,.38	12.31 (11.95 ± 0.36)

[a] The PCE values were calculated from 20 devices for each case.

Table S3. Photovoltaic performances based on **PTB7-Th**:**3TT-FIC**:**$PC_{71}BM$** devices with different DIO contents under a standard illumination (AM 1.5 G, 100 mW cm^{-2}).

DIO [vol%]	V_{oc} [V]	FF	J_{sc}^{CV} [mA cm^{-2}]	PCE [%][a)]
0	0.641	0.653	25.24	10.56 (10.38 ± 0.18)
0.4	0.657	0.694	26.63	12.14 (11.83 ± 0.31)
0.8	0.665	0.714	27.51	13.06 (12.71 ± 0.35)
1.0	0.669	0.730	27.73	13.54 (13.33 ± 0.21)
1.2	0.674	0.721	26.95	13.10 (12.83 ± 0.27)

[a)] the PCE value was calculated from 20 devices

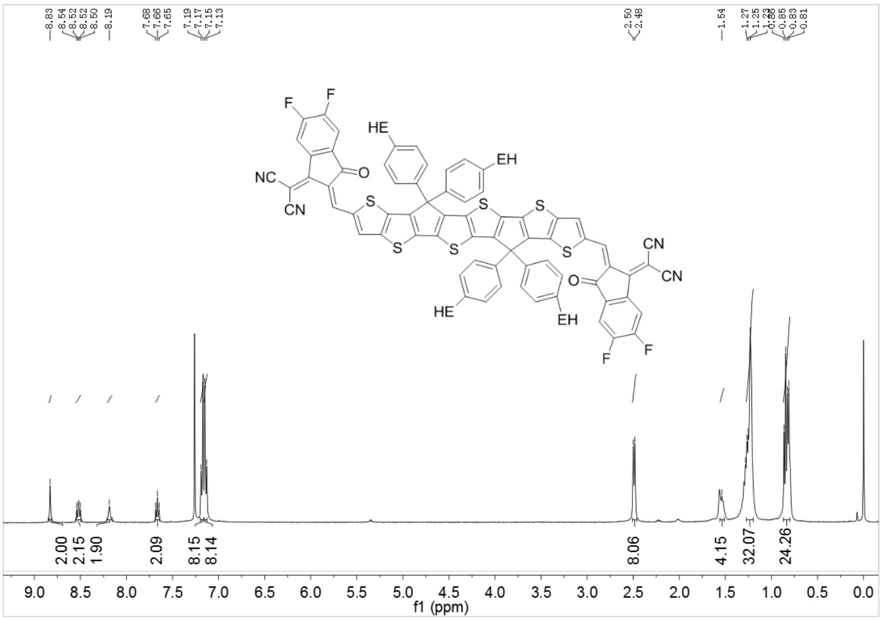

Figure S9. ^{1}H NMR of compound **3TT-FIC** in $CDCl_3$

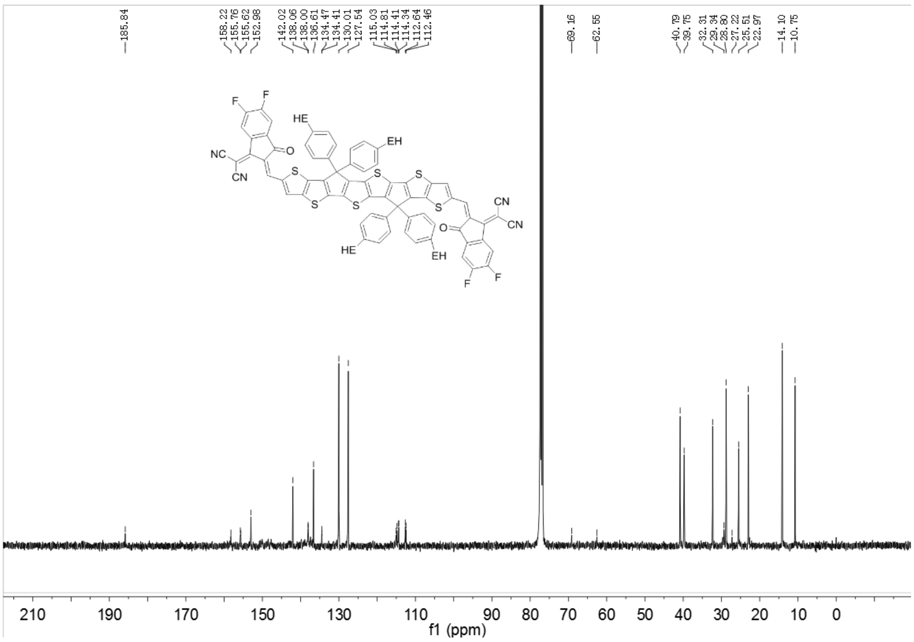

Figure S10. ^{1}C NMR of compound **3TT-FIC** in $CDCl_3$.

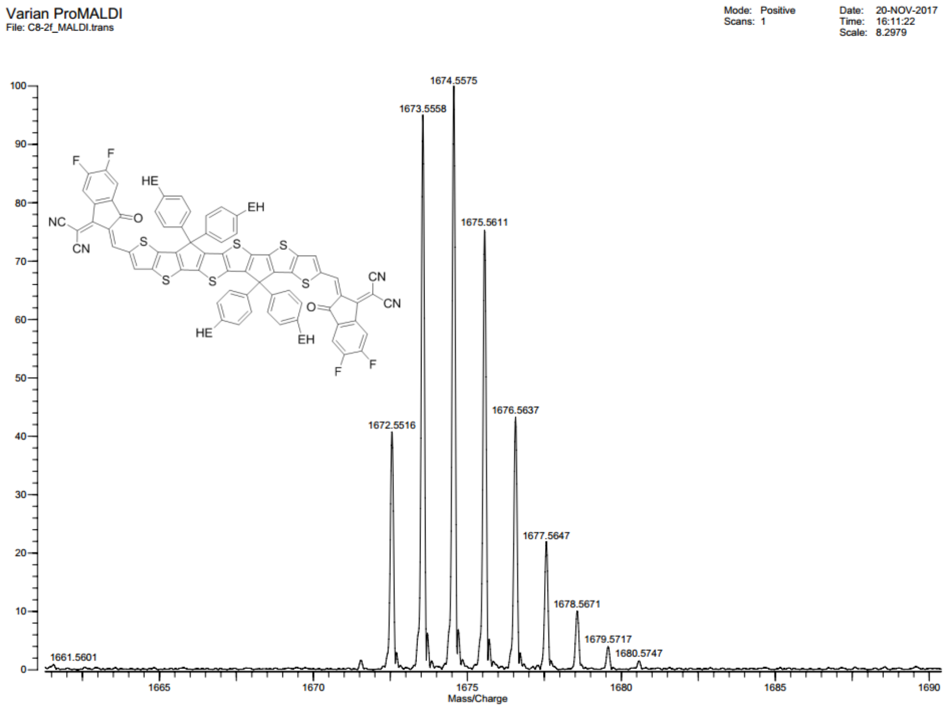

Figure S11. HR-MS spectrum of compound 3**TT-FIC**.

Reference

1. Gaussian 09, Revision B.01, M. Frisch, G. Trucks, H. Schlegel, G. Scuseria, M. Robb, J. Cheeseman, G. Scalmani, V. Barone, B. Mennucci, G. Petersson, H. Nakatsuji, M. Caricato, X. Li, H. Hratchian, A. Izmaylov, J. Bloino, G. Zheng, J. Sonnenberg, M. Hada, M. Ehara, K. Toyota, R. Fukuda, J. Hasegawa, M. Ishida, T. Nakajima, Y. Honda, O. Kitao, H. Nakai, T. Vreven, J. Montgomery, Jr., J. Peralta, F. Ogliaro, M. Bearpark, J. Heyd, E. Brothers, K. Kudin, V. Staroverov, T. Keith, R. Kobayashi, J. Normand, K. Raghavachari, A. Rendell, J. Burant, S. Iyengar, J. Tomasi, M. Cossi, N. Rega, J. Millam, M. Klene, J. Knox, J. Cross, V. Bakken, C. Adamo, J. Jaramillo, R. Gomperts, R. Stratmann, O. Yazyev, A. Austin, R. Cammi, C. Pomelli, J. Ochterski, R. Martin, K. Morokuma, V. Zakrzewski, G. Voth, P. Salvador, J. Dannenberg, S. Dapprich, A. Daniels, O. Farkas, J. Foresman, J. Ortiz, J. Cioslowski, D. Fox, Gaussian, Inc., Wallingford CT, (2010).
